# Spatial signalling mediated by the transforming growth factor-β signalling pathway during tooth formation

**DOI:** 10.1038/ijos.2016.45

**Published:** 2016-12-16

**Authors:** Xin-Yu He, Ke Sun, Ruo-Shi Xu, Jia-Li Tan, Cai-Xia Pi, Mian Wan, Yi-Ran Peng, Ling Ye, Li-Wei Zheng, Xue-Dong Zhou

**Affiliations:** 1 State Key Laboratory of Oral Diseases, West China Hospital of Stomatology, Sichuan University, Chengdu, China; 2 West China School of Stomatology, Sichuan University, Chengdu, China; 3 Guanghua School of Stomatology, Hospital of Stomatology, Department of Endodontics, Guangdong Provincial Key Laboratory of Stomatology, Sun Yat-sen University, Guangzhou, China

**Keywords:** positional information, transforming growth factor-β signalling pathway, tooth development

## Abstract

Tooth development relies on sequential and reciprocal interactions between the epithelial and mesenchymal tissues, and it is continuously regulated by a variety of conserved and specific temporal-spatial signalling pathways. It is well known that suspensions of tooth germ cells can form tooth-like structures after losing the positional information provided by the epithelial and mesenchymal tissues. However, the particular stage in which the tooth germ cells start to form tooth-like structures after losing their positional information remains unclear. In this study, we investigated the reassociation of tooth germ cells suspension from different morphological stages during tooth development and the phosphorylation of Smad2/3 in this process. Four tooth morphological stages were designed in this study. The results showed that tooth germ cells formed odontogenic tissue at embryonic day (E) 14.5, which is referred to as the cap stage, and they formed tooth-like structures at E16.5, which is referred to as the early bell stage, and E18.5, which is referred to as the late bell stage. Moreover, the transforming growth factor-β signalling pathway might play a role in this process.

## Introduction

Many elegantly designed studies have shown that tooth development in mammals and other vertebrates is the result of tightly regulated interactions between the dental epithelium and the dental mesenchyme.^1, 2, 3^ Epithelium–mesenchyme-based tissue-engineering approaches for creating artificial teeth are urgently needed. Numerous molecular and cellular studies have established that the dental epithelium and mesenchyme can form tooth-like tissues.^4, 5^ Furthermore, the recombination of either intact, primordial epithelial and mesenchymal tissues or their cell suspensions can form tooth-like tissues. The dissociated tissues from the third molar tooth buds of pigs can recombine to form both dentin and enamel.^6^ Cell suspensions harvested from the tooth buds of embryonic day (E) 14.5 mice and seeded onto collagen scaffolds can also form functional teeth.^7, 8^ In addition to embryonic tooth bud suspensions, postnatal tooth buds can also form tooth-like tissues.^9^

The transforming growth factor (TGF)-β signalling pathway plays an important role in a broad range of cellular processes. Genetically modified mouse models have confirmed that cell-autonomous TGF-β signalling is required for tooth formation.^10^ Recent studies have suggested that the TGF-β signalling pathway is involved in tooth root development^11^ and alveolar bone osteogenesis.^12^ However, whether the TGF-β pathway participates in the formation of bioengineered teeth remains unknown.

To date, the vast majority of successful tooth recombination studies have used intact dental tissues or suspensions of dental tissues. Although extremely informative, these studies have not identified the stage in which the tooth germ cells can form tooth-like structures after the loss of positional information from the epithelial and mesenchymal tissues. To overcome this obstacle, we examined single-cell suspensions from different morphological stages and compared them with untreated tooth germ. Furthermore, we investigated the activation of the TGF-β signalling pathway by examining the phosphorylation of Smad2/3. Our results demonstrated that single cells from tooth germ formed odontogenic tissue at E14.5, the cap stage, and formed tooth-like structures at E16.5 and E18.5, the bell stage. Moreover, the TGF-β signalling pathway might be one of the spatial signals involved in tooth formation.

## Materials and methods

### Isolation and preparation of tooth germ from foetal rats

All experiments involving the use of animals were reviewed and approved by the Chengdu Dossy Biological Technology. All tissues were collected in accordance with the guidelines issued by Sichuan University. The study and consent procedures were approved by the ethical committees of the West China School of Stomatology, Sichuan University and the State Key Laboratory of Oral Diseases. Pregnant rats were killed with excessive anaesthesia, and the E13.5–E18.5 rats were harvested and sanitized with 1 000 U·mL^−1^ penicillin and 1 mg·mL^−1^ streptomycin. The first molar tooth germs were carefully dissociated from the prepared mandibles and washed in phosphate-buffered saline (PBS) with 100 U·mL^−1^ penicillin and 0.1 mg·mL^−1^ streptomycin.

### Preparation of single-cell suspensions

All cells were prepared as previously described.^7^ Briefly, the first molar tooth germs were carefully harvested from the mandibles of E13.5–E18.5 rats. The tooth germs were then individually minced into <1 mm^3^ pieces, washed in PBS, enzymatically digested with 0.3 mg·mL^−1^ of type I collagenase (17100-017; Gibco, Grand Island, NY, USA) and 0.4 mg·mL^−1^ of Dispase I (17105-041; Gibco, Grand Island, NY, USA) for 45 min at 37 °C, and gently dissociated by trituration. Cells were washed and filtered using a sterile Falcon 40-micron cell strainer (Corning, Tewksbury, MA, USA) to generate single-cell suspensions.

### Preparation of reassociated constructs and transplantation under renal capsules

The cell-loaded constructs of single-cell suspensions consisted of 1.0 × 10^6^ cells suspended in 50 μL of 1 mg·mL^−1^ of collagen type I (2.0 × 10^4^ cells per μL). The constructs were suspended in collagen and gelled at 37 °C for 1 h before being transplanted under the renal capsules. Laparotomies were performed on rats, and the reassociated constructs were implanted in the renal capsules as previously described.^13, 14^ The numbers of the single-cell suspension constructs were as follows: E13.5(4/6), E14.5(4/5), E16.5(4/6), and E18.5(5/8). The numbers of the tooth germ transplants were as follows: E13.5(3/3), E14.5(3/4), E16.5(3/4), and E18.5(3/3).

### Haematoxylin and eosin staining, Masson's trichrome staining and immunohistochemistry staining of reassociation constructs

Paraffin-embedded samples were sectioned at a thickness of 5 μm. Selected sections were then stained with haematoxylin and eosin (H&E) and analysed under bright-field microscopy (BX53; Olympus, Tokyo, Japan). For Masson's trichrome staining, we used Weigert's iron haematoxylin, Biebrich scarlet-acid fuchsin and aniline blue. Immunohistochemical analyses were performed on selected sections of each construct using a tooth-specific antibody against amelogenin (sc-32892; Santa Cruz Biotechnology, Paso Robles, CA, USA), a TGF-β pathway-specific antibody against p-Smad2/3 (sc-11769; Santa Cruz Biotechnology, Paso Robles, CA, USA) and an antibody against smad2/3 (NBP1-19520; Novus Biologicals, Littleton, CO, USA). For immunohistochemistry (IHC), all sections were incubated with a biotinylated secondary antibody, stained using the R&D HRP-DAB staining kit (R&D Systems, Minneapolis, MN, USA) and counterstained with haematoxylin. Processed sections were dehydrated through a series of graded ethanol baths, sealed with Permount (Thermo Fisher Scientific, Waltham, MA, USA), and analysed using bright-field microscopy. Photographs were obtained with a digital camera and manipulated using Adobe Photoshop.

### Statistical analysis

Significance (*P*<0.05) was determined using the unpaired Student's *t*-test and analysed using the SPSS 16.0 software package.

## Results

### Morphological characterization of the transplants

**Figure 1:**
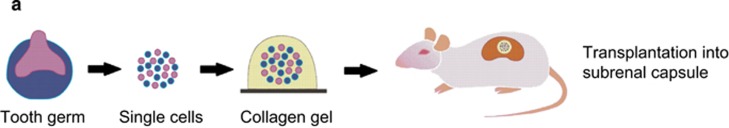

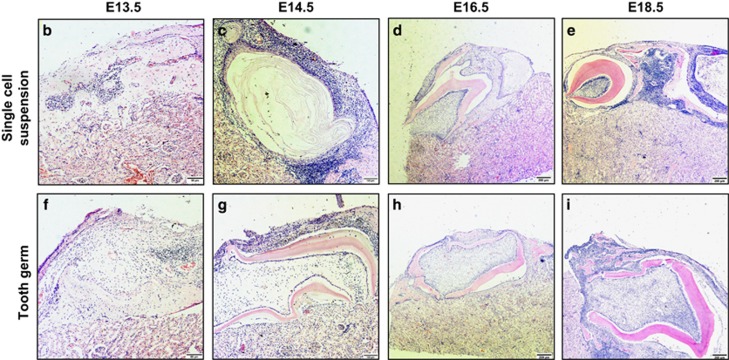
**Morphological analysis of the transplants**. (**a**) Schematic diagram of the reaggregated system. (**b**) Recombination from an E13.5 single-cell suspensions appeared as disordered tissue on an H&E-stained section. (**c**) At E14.5, the single-cell suspensions formed several epithelial pearls. (**d**, **e**) At E16.5 and E18.5, the single-cell suspensions exhibited tooth-like tissue. (**f**) At E13.5, the tooth germ transplants formed a boundary between the epithelial and mesenchymal tissue. (**g**–**i**) And at E14.5, E16.5 and E18.5, the tooth germ formed tooth-like tissue. Scale bar for **b** and **f**: 50 μm; for **c** and **g**: 100 μm; and for **d**, **e**, **h** and **i**: 200 μm. E, embryonic day.

**Figure 2:**
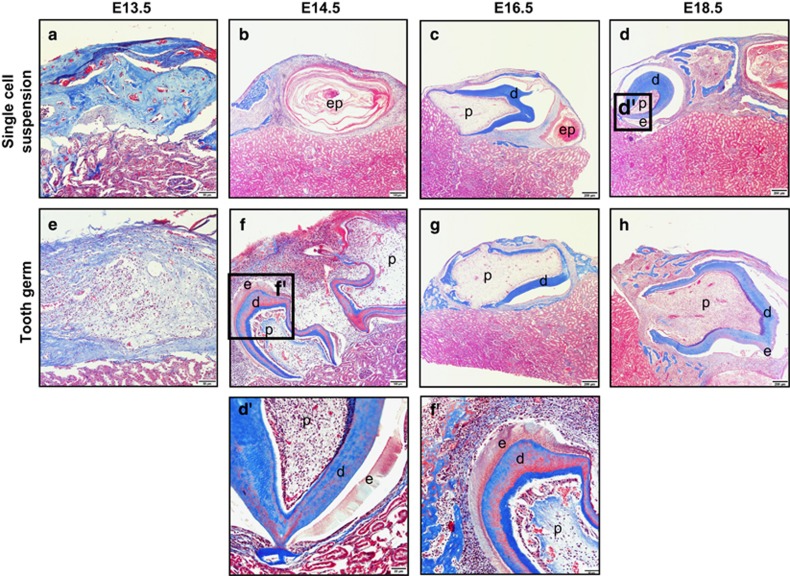
**Histological analysis of transplants**. (**a**) At E13.5, the single-cell suspensions had no tissue boundary. (**b**) At E14.5, the single-cell suspensions formed several epithelial pearls. (**c**) At E16.5, pulp tissue, dentin and apical constriction were observed, but no enamel was observed. (**d**) At E18.5, the single-cell suspensions started to form immature enamel tissue. (**e**) The tooth germ transplants formed a boundary between the epithelial and mesenchymal tissue. (**f**, **h**) Pulp tissue, dentin, apical constriction and immature enamel tissue were observed in both the E14.5 and E18.5 suspensions, (**g**) but no enamel was observed in the E16.5 suspensions. Scale bar for **a** and **e**: 50 μm; for **b** and **f**: 100 μm; for **c**, **d**, **g** and **h**: 200 μm; for **d**': 20 μm; and for **f**': 50 μm. E, embryonic day.

All cells were prepared and transplanted into the renal capsules as described in the Materials and Methods section (Figure 1a). After sample collection, all transplants were separated from the host renal capsules, embedded in paraffin and sectioned. Morphological analysis by HE staining was performed to examine the stage in which the single-cell suspensions started to form tooth-like structures. The reassociation of the E13.5 single-cell suspensions was characterized by disordered tissue that failed to organize (Figure 1b). At E14.5, the single-cell suspensions had formed several epithelial pearls, but no tooth-like structures were observed (Figure 1c). At E16.5 and E18.5, the single-cell suspensions had formed tooth-like tissues (Figure 2d and 2e). The E13.5 tooth germ transplants presented clear boundaries between the epithelial tissues and the mesenchymal tissues (Figure 1f), similar to the results of other studies.^13^ At E14.5, E16.5 and E18.5, the transplants formed tooth-like tissues, including dental pulp and dentin, and exhibited apical constriction (Figure 1g–1i). This finding indicates that E16.5 might be the earliest stage in which tooth-like structures can form after the loss of positional information from the epithelial and mesenchymal tissues.

### Histological characterization of the transplants

Masson's trichrome staining showed that there was no boundary between the epithelial tissue and mesenchymal tissue in the E13.5 single-cell suspensions (Figure 2a). Epithelial pearls were observed in both the E14.5 and E16.5 single-cell suspensions (Figure 2b and 2c). In addition, single-cell suspensions from E16.5 exhibited pulp tissue, dentin and apical constriction, but no enamel tissue. We also observed immature enamel tissue adjacent to the dentin at E18.5 (Figure 2d and 2d'). The tooth germ transplants started to exhibit clear histological layers at E14.5 (Figure 2e). We observed pulp tissue, dentin, apical constriction and immature enamel tissue in both the E14.5 and E18.5 transplants (Figure 2f, 2f' and 2h), but no enamel was observed in the E16.5 transplants.

### Immunocytochemical analysis of the transplants

**Figure 3:**
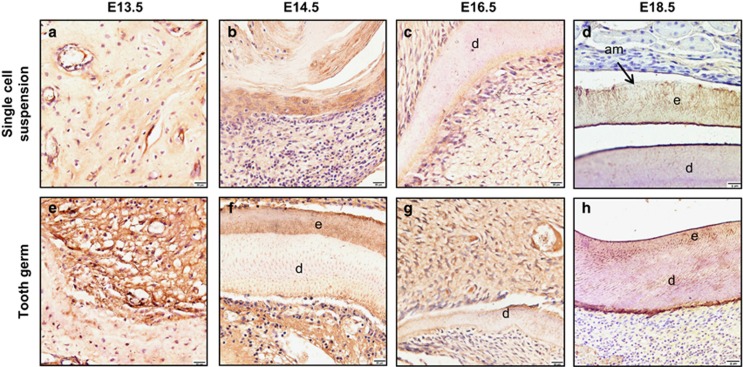
**Immunohistochemical analysis of amelogenin expression**. (**a**–**c**) At E13.5, E14.5 E16.5, the single-cell suspensions has no obvious positive staining of amelogenin. (**d**) The single-cell suspensions started to exhibit positive staining at E18.5. (**f**, **h**) The tooth germ transplants revealed a clear epithelial–mesenchymal boundary at E13.5. (**f**, **h**) The amelogenin-positive staining could also be detected at E14.5 and E18.5. (**g**) However, there has no obvious positive staining at E16.5. Scale bar for **a**–**g**: 20 μm and for **d** and **h**: 8 μm. E, embryonic day.

We examined the expression of amelogenin using IHC. The single-cell suspensions started to exhibit positive staining at E18.5 (Figure 3d), and it closely resembled decalcified enamel adjacent to the dentin. The tooth germ transplants revealed a clear epithelial–mesenchymal boundary at E13.5 (Figure 3e), and amelogenin-positive staining was also detected in the matrix at E14.5 and E18.5 (Figure 3f and 3h).

### TGF-β signalling in transplants

**Figure 4:**
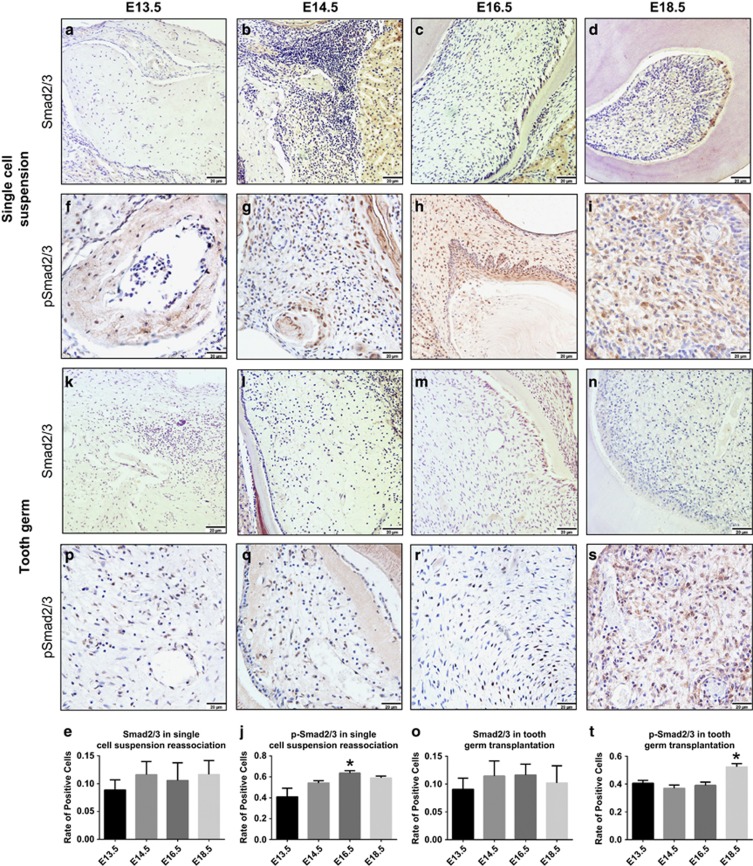
**Immunohistochemical analysis of the expression and phosphorylation of Smad2/3**. The Smad2/3- and p-Smad2/3-positive cells were counted in each view, and the rate of positively stained cells was determined. (**a**–**e**, **k**–**o**). Smad2/3 (stained brown) expression was analysed in the cell cytoplasm. The rate of cells positively stained for Smad2/3 was the same across all of these stages. Scale bar: 20 μm. (**f**–**j**) p-Smad2/3 expression (brown staining) was analysed in the cell nucleus. The single-cell suspensions had a higher rate of positive cells at E16.5. (**p**–**t**) However, the tooth germ had a higher rate of positive cells at E18.5. All data are presented as the mean±standard deviation, *P<0.05 by unpaired *t*-test. Scale bar: 20 μm. E, embryonic day.

In response to activation of the TGF-β signalling pathway, Smad2 and Smad3 are phosphorylated and translocated from the cytosol to the nucleus.^15, 16^ To investigate whether the TGF-β signalling pathway was involved in the reassociation process, we examined the phosphorylation of Smad2/3 using IHC. The rate of positively stained cells in the single-cell suspensions from E16.5 was higher than in the other stages (Figure 4f–4j). However, the rate of positively stained cells in the tooth germ transplants was the same at E13.5, E14.5 and E16.5 (Figure 4p–4r). In addition, the rate of positively stained cells at E18.5 was higher than the other groups (Figure 4s and 4t). We also examined the expression of nonphosphorylated Smad2/3 as a control using IHC (Figure 4a–4e and 4k–4o). This finding indicates that the TGF-β signalling pathway may play a role in tooth development.

## Discussion

In this study, we investigated the stages at which tooth germ cell suspensions can form tooth-like tissues after losing their positional information. As other studies have reported,^8^ we showed that successful odontogenic tissue (that is, epithelial pearls) formed during the cap stage (E14.5), while tooth-like tissues, dentin and apical constriction started to develop during the early bell stage (E16.5). In addition, enamel appeared in the late bell stage (E18.5). These findings suggest that single-cell suspensions of later morphological stages may form more mature, tooth-like structures. Moreover, the phosphorylation level of Smad2/3 was higher in both single-cell suspensions at E16.5 and tooth germ transplants at E18.5. These results indicate that the TGF-β pathway may be involved in this tooth development.

It is possible to bioengineer teeth from embryonic tooth bud cells,^17, 18^ and even postnatal tooth buds can form tooth-like tissues.^9^ In previous studies, the epithelium and mesenchyme were collected and suspended individually before being reassociated, and the relative orientation of the epithelium and mesenchyme was largely maintained. The results showed that the reassociation of E14 dental epithelium and dental mesenchyme single-cell suspensions resulted in the formation of a bioengineered tooth unit that included the developing root, differentiated odontoblasts, cementum and periodontal ligament fibroblasts, with the development of the root surface and newly formed bone occurring after *in vivo* transplantation.^19^ An analysis of 12-week-old implant tissue from dissociated rat tooth bud cells that were 4 days postnatal also demonstrated that suspensions from tooth buds can reliably generate bioengineered tooth tissue with roots and bones.^4^ Our study involved tooth buds that were suspended as complete units, and the positional information was completely disrupted. In this situation, the tooth bud can form a tooth-like structure.

Previous studies have suggested that the SHH, FGF, NOTCH, BMP and WNT signalling pathways are involved in the regulation of tooth histogenesis, morphogenesis and cell differentiation.^20, 21, 22^ The TGF-β pathway is involved in many cellular processes in both mature organisms and embryos, including cell proliferation, cell differentiation and other cellular functions.^23^ There is also extensive crosstalk between the TGF-β pathway and other signalling pathways, such as BMP.^24, 25, 26^ Although few studies have addressed the involvement of the TGF-β pathway in dental development, some evidence suggests that TGF-β plays a role in this process. Tgfbr2-deficient mice exhibited malformed incisors with wavy mineralized structures, and this phenotype may be caused by an upregulation of Wnt5a expression and a downregulation of Fgf3/10 expression in the mesenchyme.^27^ Moreover, Hertwig's epithelial root sheath cells are strongly positive for TGF-β1. In addition, positive staining for p-Smad2/3 has been observed in bone and periodontal ligaments.^28^ Taken together, our results and the above studies indicate that the TGF-β signalling pathway may play a role in tooth formation.

The ultimate goal of bioengineering studies is to develop regenerative therapies that will restore lost or damaged teeth.^29, 30^ Previous studies of three-dimensional organ cultures revealed that embryonic tooth cells can generate bioengineered organs that are fully functional. In our study, we found that single tooth germ cells started to form tooth-like structures at the early bell stage (E16.5) after losing their positional information. Further studies using seed cells may confirm our study findings and result in a greater understanding of the clinical applications for bioengineered teeth in regenerative therapy.

## Conclusion

This study revealed the following important findings: after the loss of tissue positional information, single-cell suspensions of tooth germ cells formed epithelial pearls at the cap stage (E14.5) and formed tooth-like structures at E16.5 and E18.5. In addition, the TGF-beta signalling pathway might play a role in this process.
